# Publisher’s Note: Continued Publication of *Neurology International* by MDPI

**DOI:** 10.3390/neurolint12030008

**Published:** 2020-10-16

**Authors:** Shu-Kun Lin, Franck Vazquez, Unai Vicario

**Affiliations:** 1 MDPI, St. Alban-Anlage 66, CH-4052 Basel, Switzerland; lin@mdpi.com; 2 MDPI, Avenida Madrid, 95, 08028 Barcelona, Spain; vicario@mdpi.com

*Neurology International* was launched in 2009 and it has been published over the past eleven years by PAGEPress Publications [1]. Dr. Michael A. Meyer has served as its Editor-in-Chief since its inception [2] and is currently still active in this role.

We are delighted to take over the publication of *Neurology International* from PAGEPress and continue the legacy of this journal, and ensure that we serve well the neurosciences communities. *Neurology International* complements very well the MDPI portfolio of neurosciences journals, especially *Neuroglia* [3], *NeuroSci* [4] and *Brain Sciences* [5].

MDPI will publish the fourth quarterly issue of 2020, and thereafter publish four quarterly issues as of 2021.

Looking forward to publishing your research work in *Neurology International* [6]!
